# Abstracts

**DOI:** 10.1155/S1463924685000372

**Published:** 1985


Journal of Automatic Chemistry/*Journal of Clinical Laboratory Automation*, Vol. 7, No. 4 (October–December 1985), pp. 170–172

### Abstracts

### Page 173

**A laboratory and in-ward evaluation of cholesterol assays on the Ames Seralyzer**
L. M. Nelson *et al.*

The Ames Seralyzer was assessed both in the laboratory and in the coronary care unit to determine whether the performance characteristics would allow cholesterol assays to be done, with clinical advantage, close to the patient. In the laboratory evaluation, using modern objective criteria of acceptability, linearity was statistically satisfactory, but imprecision and inaccuracy were unsatisfactory. The performance achieved by three junior doctors was significantly worse than that attained in the laboratory. The instrument was therefore considered to be unsuitable for use both within and outside the laboratory for cholesterol assays, except perhaps for first line detection of hypercholesterolaemia.

*This article was rushed through press and so French and German translations of the abstract will be published in the January–March 1986 issue.*

---

### Page 177

**Automated preparation of biological samples prior to high pressure liquid chromatography: Part I – The use of dialysis for deproteinizing serum for amino-acid analysis**
D. C. Turnell and J. D. H. Cooper

An automatic system for the preparation and analysis of amino-acids in biological samples is described. The procedure incorporates dialysis as a means for deproteinizing samples prior to the estimation of the amino-acids using pre-column derivatization with orthphthaladehyde/2-mercaptoethanol and reverse phase high pressure liquid chromatography (HPLC). The system performs sampling, dialysis and derivatization reactions in 135 s and the efficiency of the clean-up technique extends analytical column life. Within-run coefficients of variation ranging from 1.1–3.8% were observed for different amino-acids.

**Préparation automatisée d'échantillons biologiques pour la chromatographie liquide à haute pression: l'utilisation de la dialyse pour déprotonéiser le sérum pour l'analyse d'acides aminés**

D. C. Turnell and J. D. H. Cooper

Un système automatique pour la préparation et l'analyse d'acides aminés dans des échantillons biologiques est décrit. La procédure incorpore la dialyse pour déprotonéiser les échantillons avant la séparation des acides aminés utilisant une colonne pour la dérivatisation et la chromatographie liquide à haute pression et phase renversée (HPLC). Le système exécute l'échantillonnage, la dialyse et la réaction de dérivatisation en 135 secondes. L'efficacité de cette procédure rallonge la durée de vie de la colonne analytique. Les coéfficients de variation en une série pour les différents acides aminés se situaient entre 1.1–3.8%.

**Automatische Herstellung biologischer Proben vorgängig zur HPLC. Teil 1: Die Anwendung der Dialyse zur Entproteinisierung von Serum für die Aminosäuren-Analyse**
D. C. Turnell and J. D. H. Cooper

Es wird ein automatisches System für die Herstellung und Analyse von Aminosäuren in biologischen Proben beschrieben. Das Prozedere umfasst Dialyse als Mittel für die Entproteinisierung von Proben vorgängig der Bestimmung der Aminosäuren mit Vorkolonnen-Derivatisierung und Reverse Phase HPLC. Das System führt die Probenahme, Dialyse und Derivatisierungs-Reaktion in 135 s durch und die Wirksamkeit der Waschtechnik übersteigt die analytische Lebensdauer der Kolonne. Die Variationskoeffizienten innerhalb einer Serie schwankten zwischen 1.1–3.8% für verschiedene Aminosäuren.

**Part II – The combined use of dialysis and trace enrichment for analysing biological material**
J. D. H. Cooper and D. C. Turnell

A new technique for the automatic preparation of biological samples prior to high pressure liquid chromatography (HPLC) analysis is described. The procedure combines the use of trace enrichment and dialysis and offers advantages over the conventional manual and automatic extraction procedures presently employed. The estimation of phenobarbitone in human serum is used to examine the potential of the technique. A within-run coefficient of variation of 1.4% was obtained.

**Préparation automatique d'échantillons biologiques pour la chromatographie liquide à haute pression: deuxième partie: l'utilisation combinée de la dialyse et de la concentration pour l'analyse de matériel biologique**

J. D. H. Cooper and D. C. Turnell

Une nouvelle technique pour la préparation automatique d'échantillons biologiques pour la chromatographie liquide à haute pression (HPLC) est décrite. Le procédé combine l'utilisation d'enrichissement de traces et la dialyse et offre des avantages comparés aux procédés d'extraction manuelle et automatiques utilisés présentement. Le dosage du phénobarbitone dans le sérum humain est utilisé pour examiner le potentiel de cette technique. Un coéfficient de variation de 1.4% a été obtenu.

**Automatische Herstellung biologischer Proben vorgängig zur HPLC. Teil 2: Kombination von Dialyse und Spurenanreicherung für die Analyse biologischen Materials**
J. D. H. Cooper and D. C. Turnell

Es wird eine neue Technik für die automatische Herstellung biologischer Proben vorgängig der Analyse mit HPLC beschrieben. Das Prozedere kombiniert die Anwendung der Analyse mit der Spurenanreicherung und ist gegenüber konventionellen und automatischen Extraktionsvorgängen, wie sie heute verwendet werden, vorteilhaft. Die Bestimmung von Phenobarbiton in menschlichem Serum wird benützt, um das Potential dieser Technik zu ergründen. Für den Variationskoeffizienten innerhalb einer Messreihe wurde 1.4% erhalten.

---

### Page 185

### Laboratory information management – the CALS approach
J. Boother

The CALS Lab Manager System is a laboratory information management tool which facilitates the efficient and accurate flow of data within the laboratory environment. This paper gives a system overview and details of how key procedures in a laboratory are performed.

### La gestion de l'information au laboratoire – l'approche CALS
J. Boother

Le système de gestion de laboratoire CALS est un instrument de gestion d'informations au laboratoire qui facilite le flux efficace et précis des données à l'intérieur de l'environnement de laboratoire. Cet article donne un aperçu du système et des détails comment fonctionnent les procédés principaux dans le laboratoire.

### Das CALS Laborinformationssystem

J. Boother

Das CALS Laborführungssystem ist ein Werkzeug zur effizienten und sicheren Handhabung von Labordaten. Die vorliegende Arbeit gibt eine Uebersicht dieses Systems sowie einige Details zur Art wie Schlüsselprozeduren in einem Labor durchgeführt werden.

---

### Page 192

### Microcomputers in a water authority laboratory
M. P. Bertenshaw and K. C. Wheatstone

Water authorities must analyse large numbers of samples from all areas of the hydrological cycle in order to monitor and maintain required standards of water quality in potable supplies, rivers and effluents. For example, Severn-Trent Water Authority, one of 10 in England and Wales, carries out about 2·5 M chemical and bacteriological determinations per year. The data management system must, therefore, provide the basis for automation within a water authority laboratory and also provide facilities to report results outside the laboratory. This paper demonstrates areas in which microcomputers have been used to provide a bridge between commercially available instrumentation for both automated and manual water analysis and a laboratory data management system. Benefits of automatic data-capture and areas for future development are discussed.

*French and German abstracts will be published in our next issue.*

---

### Page 197

### Haemoglobin analysis on whole blood by reflectance photometry
J. A. Lott and E. Khabbaza

For whole blood haemoglobin analysis, the Ames test strips were used in the Ames Seralyzer. Precise results and an average coefficient of variation of 2% over the range of approximately 8 g/dl to 20 g/dl haemoglobin were achieved. Three test sites were utilized for this study. All analyses were done on fresh blood specimens, and the Ames method was compared with the Coulter-S method. The Ames method agreed very well with the Coulter-S method; however, above 18 to 20 g/dl, there is some bias between the above two methods, with the Seralyzer generally giving higher results. Bilirubin, carboxyhaemoglobin, and lipaemia did not interfere with the Ames procedure.

The Seralyzer is easy to use, but careful pipetting of the specimen is a critical step. Calibration is performed with a stable dye solution. The approximate 1 min/test output makes the Seralyzer suitable for routine use in clinical laboratories.

### L'analyse de l'hémoglobine dans le sang par photometrie de réflexion
J. A. Lott and E. Khabbaza

Pour l'analyse de l'hémoglobine dans le sang des rubans de test Ames sont utilisés dans le Seralyzer Ames. Des résultats précis et un coéfficient de variation moyen de 2% ont été obtenus pour le domaine entre 8 g/dl et 20 g/dl. Trois sites de mesure ont été utilisés pour cette étude. Toutes les analyses ont été éffectuées avec des specimens de sang frais et la méthode Ames à été comparée avec la méthode Coulter-S. La méthode Ames correspond très bien avec la méthode Coulter-S, cependant en dessous de 18 à 20 g/dl on trouve une légère différence entre ces deux méthodes, le Seralyzer donnant des résultats légèrement plus élevés. La bilirubine, le carboxyhémoglobine et lipémie n'interfèrent pas avec le procédé Ames.

Le Seralyzer est facile à l'usage cependant le pipettage des échantillons est un pas critique. Le calibrage est fait avec une solution colorée stable. Le temps d'analyse typique de 1 minute par mesure rend le Seralyzer convenable pour l'utilisation de routine dans les laboratoires cliniques.

### Hämoglobin-Analyse von Gesamtblut mittels Reflexionsphotometrie
J. A. Lott and E. Khabbaza

Für die Hämoglobin-Analyse von Gesamtblut wurden Ames Teststreifen im Ames Seralyzer verwendet. Es wurden präzise Resultate und ein durchschnittlicher Variationskoeffizient von 2% über den Bereich von 8 g/dl bis 20 g/dl Hämoglobin erreicht. Drei Testorte wurden für diese Studie verwendet. Alle Analysen wurden an frischen Blutproben durchgeführt, und die Ames-Methode wurde mit der Coulter-S-Methode verglichen. Die Ames-Methode stimmte gut mit der Coulter-S-Methode überein; zwischen 18 und 20 g/dl tritt jedoch eine Abweichung zwischen den beiden Methoden auf, wobei der Seralyzer im allgemeinen höhere Resultate ergibt. Bilirubin, Carboxyhämoglobin und Lipämia interferieren nicht mit dem Ames-Prozedere.

Der Seralyzer ist leicht zu benützen, aber das sorgfältige Pipettieren der Probe ist ein kritischer Schritt. Die Eichung wird mit einer stabilen Farblösung durchgeführt. Der ungefähre Durchsatz von einer Probe pro Minute macht den Seralyzer geeignet für Routine-Untersuchungen im klinischen Labor.

---

**Page 201**

**High performance ion-exchange chromatography of amino-acids in biological fluids using Chromakon 500 – performance of the apparatus**
L. Cynober *et al.*

The analytical performance of a high pressure ion-exchange chromatography system (Kontron's Chromakon 500) for amino-acid measurements in biological fluids was tested. 39 amino-acids and derivatives were separated. Retention times were constant, with CVs ranging from 0·1% to 1·3%. Reaction was linear to at least 1250 μmol/l for all amino-acids. Detection limits were generally 5 μmol/l. CVs of repeatability assay were less than 5%, except for phenylalanine (8·3%). Mean reproductibility was 10·2%. The Chromakon 500 thus appears to provide satisfactory results for all amino-acids rapidly, i.e. 170 min including regeneration time.

*French and German abstracts to follow.*

---

**The following are French and German summaries of papers published in the previous (Vol. 7, No. 3, July–September) issue:**

**Page 130**

**Automation en chromatographie à phase gazeuse multidimensionelle**
G. M. Ogle *et al*

Le développement du microprocesseur et son usage dans l'instrumentation analytique à mené à de grandes améliorations en performance. Ces améliorations sont faciles a voire dans le domaine de la chromatographie en phase gazeuse.

La chromatographie en phase gazeuse multidimensionelle est une technique dans laquelle plus d'une colonne est utilisée dans le système analytique mènant à une très haute résolution des composantes de l'échantillon. Beaucoup dedemandes sont requises dans l'instrumentation par l'utilisation de la chromatographie multidimensionelle et celles-ci sont illustrées par la construction d'un chromatograph en phase gazeuse récemment mise sur le marché.

Un system multidimensionel 'heart cut' est décrit en détail et son application à l'analyse des alcools dans le pétrol est discuté. Due aux exigences de l'environnement l'utilisation des alkyls de plomb pour augmenter la valeure octane dans l'essence est en voie de disparition et les alcools bas de $C_2$ à $C_5$ sont utilisés plus fréquemment. Utilisant la chromatographie multidimensionelle le temps d'analyse est d'environ 10 minutes au lieu de 1 heure avec une seule colonne capillaire, ceci permettant d'analyser un nombre nettement plus élevé d'échantillons par jour.

**Automation in multidemensionaler Gaschromatographie**
G. M. Ogle *et al.*

Die Entwicklung des Mikroprozessors und seine Verwendung in analytischer Instrumentierung hat zu vielen Leistungsverbesserungen geführt. Diese Verbesserungen werden am Beispiel der Gaschromatographie illustriert.

Multidimensionale Gaschromatographie ist eine Technik, wo mehr als eine Kolonne im analytischen System verwendet wird, um damit eine hohe Auflösung der Komponenten in der Probe zu erzielen. Viele Anforderungen werden gestellt an die Instrumentierung bei Verwendung der multidimensionalen Chromatographie und diese werden am Beispiel der Eigenschaften eines kürzlich auf den Markt gebrachten Gaschromatographen illustriert.

Ein multidimensionales System mit Schnitt im Zentrum wird im Detail beschrieben und seine Anwendung auf die Alkoholanalyse in Benzin wird diskutiert. Aus umweltschützerischen Gründen nimmt die Zusetzung von Bleialkylen als 'anti knock' Ingredienzen zu Treibstoffen ab, und die tiefen Alkohole von $C_2$ bis $C_5$ werden zunehmend verwendet. Mit der multidimensionalen Chromatographie beträgt die Analysenzeit nur ungefähr 10 Minuten statt einer Stunde wie mit der Einzelkapillarkolonne. Damit kann der Tagesdurchsatz signifikant erhöht werden.

---

**Page 136**

**Systèmes d'information et de gestion du laboratoire**
K. J. Jones

Les systèmes d'information et de gestion du laboratoire (LIMS) ont reçus une grande attention en Grande Bretagne et d'autres pays. Cependant aucune étude indépendente du potentiel et des nécessités du marché à été effectuées jusque-la. Une étude dont les résultats sont publiés ici a été effectuée dans le cadre d'un travail pour obtenir un degré d'administration à l'école d'économie de l'université de Manchester. Cette étude essaie de qualifier le statut du LIMS dans les laboratoires du Royaume-Uni.

**Laborinformations- und Führungssysteme**

K. J. Jones

In letzter Zeit haben Laborinformations- und Führungssysteme in England und anderswo viel Aufmerksamkeit erregt. Bis jetzt wurden aber noch keine unabhängigen Untersuchungen zum Marktbedürfnis und Marktpotential durchgeführt. Eine Erhebung, deren Resultate hier publiziert sind, wurde als Teil der Anforderungen für die Erlangung des Abschlussdiplom an der University of Manchester Business School durchgeführt. Diese Erhebung versucht den Stellenwert von LIMS in den englischen Laboratorien zu qualifizieren.

